# Incidence of Cancer and Asbestos-Related Diseases among Residents Living near Abandoned Asbestos Mines in South Korea: A Retrospective Cohort Study Using National Health Insurance Database

**DOI:** 10.3390/ijerph18030875

**Published:** 2021-01-20

**Authors:** Kyeongmin Kwak, Kyung Ehi Zoh, Domyung Paek

**Affiliations:** 1 Department of Occupational and Environmental Medicine, Korea University Ansan Hospital, Ansan 15355, Korea; ggm1981@snu.ac.kr; 2 Department of Environmental Health Sciences, Graduate School of Public Health, Seoul National University, Seoul 08826, Korea; kezoh@snu.ac.kr; 3 Institute of Health and Environment, Seoul National University, Seoul 08826, Korea

**Keywords:** asbestos, environmental exposure, non-occupational exposure, asbestos mine, asbestos-related disease, cohort study, big data

## Abstract

The use of asbestos has been banned since 2009 in South Korea. However, there is still a risk of exposure to environmental asbestos originating from abandoned asbestos mines. We constructed a retrospective dynamic cohort using the National Health Insurance Database of South Korea. We determined the risk of developing asbestos-related diseases (ARDs) among residents living near asbestos mines compared with those living in the control area and the general population. The risks of asbestosis (adjusted hazards ratio [HR] 65.40, 95% CI = 35.02–122.12) and pleural plaques (adjusted HR 3.55, 95% CI = 1.96–6.41) were significantly increased among residents living near the asbestos mines compared with the control area. The risk of malignant mesothelioma was increased near asbestos mines compared with the control area; however, it was not significant (adjusted HR 1.83, 95% CI = 0.61–5.47). When a separate analysis according to sex was conducted, the risk of mesothelioma among male residents was statistically significant (adjusted HR 8.30, 95% CI = 1.04–66.63), and the standardized incidence ratio (SIR) was significantly increased (SIR 3.48, 95% CI = 1.50–6.85). The risk of ARDs was increased due to environmental asbestos exposure near abandoned asbestos mines in South Korea.

## 1. Introduction

Exposure to asbestos causes various diseases such as malignant mesothelioma, lung cancer, asbestosis, pleural effusion, diffuse pleural thickening, laryngeal cancer, and ovarian cancer [1,2,3]. Although heavy industrial exposure to asbestos is known to cause lung cancer, mesothelioma, and asbestosis, there is little evidence of how environmental exposure to asbestos causes these asbestos-related diseases (ARDs).

In South Korea, concerns about environmental exposure to asbestos and ARDs among residents living near asbestos textile factories and asbestos mines are increasing. In 2007, Kang analyzed the data on patients with malignant mesothelioma for 10 years in Busan (excluding occupational exposure) and found that the proportional hazards ratio (HR) of malignant mesothelioma was 6.5 (95% CI = 3.0–14.2) for residents who had ever lived within 500 m of asbestos textile factories compared with those who had never lived near them [4]. In addition, the Ministry of Environment (ME) conducted an epidemiological study of 215 residents within 2 km from abandoned asbestos mines in South Chungcheong Province in 2008 [5]. A total of 128 residents had no experience of working in mining, and asbestosis and pleural plaques were confirmed by chest computerized tomography in 23 and 37 of them, respectively. Based on these studies, it can be assumed that environmental ARDs may be prevalent in South Korea. 

However, these studies were cross-sectional surveys with a small number of subjects, and they were only case or exposure studies without an appropriate control group. Therefore, there is a limitation in confirming the causal relationship between environmental exposure to asbestos and ARDs. To address these limitations, we constructed a retrospective dynamic cohort using the National Health Insurance Database (NHID) of South Korea for individuals living near asbestos mines for more than five years. To identify the association between environmental exposure to asbestos and ARDs, the incidence of ARDs were compared between residents living near abandoned asbestos mines and those living in the control area and the general population.

## 2. Materials and Methods

### 2.1. Data Source

We used the NHID to conduct a retrospective cohort study from 2007 and 2018. The NHID is a public database containing data on health care utilization, health screenings, and socio-demographic variables for the entire population of South Korea, created by the Korean National Health Insurance Service (KNHIS) [6]. The NHID has been established since 2002 and includes an “eligibility database”, a “national health screening database”, and a “health care utilization database”. The eligibility database contains information on age, sex, region, income-based insurance contributions, type of insurance, and date of death. The national health screening database contains information on health behaviors such as smoking, drinking, and exercise as well as clinical laboratory results. The health care utilization database contains information on inpatient and outpatient treatment. All disease diagnoses in the NHID are described using the International Classification of Disease, Tenth Revision (ICD-10) codes. We also used the national cancer incidence data of the Korea Central Cancer Registry (KCCR) in 2007–2017. The Korean government started the KCCR in 1980 to promote the registration of cancer cases. Data from the registry are highly accurate for the diagnosis of cancer and are considered reliable in determining actual incidence rates [7,8].

### 2.2. Exposed and Control Areas

**Figure 1:**
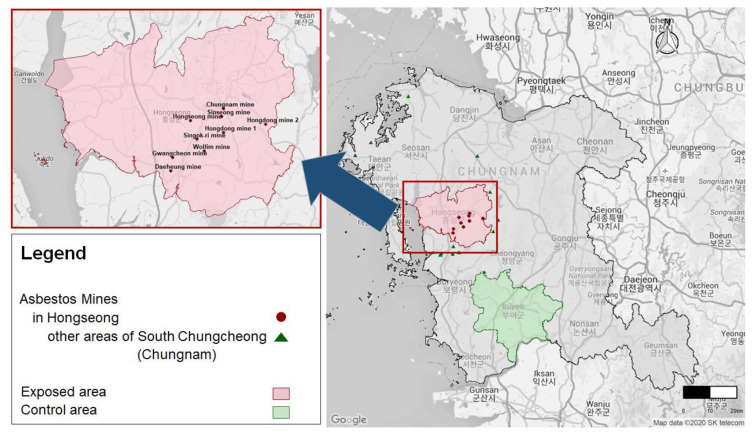
Exposed and control areas.

Among the asbestos mines in South Korea, there are 28 asbestos mines at present risk of exposure to asbestos, of which 22 mines are in South Chungcheong Province (nine mines are in Hongseong). Residence information for the subdivision (Eup-Myeon-Dong) of Hongseong was not provided by the NHID because of the protection of personal information. As around 70% of the total population in Hongseong is located within a 5 km radius of abandoned asbestos mines, Hongseong was selected as the exposed area near abandoned asbestos mines in this study (Figure 1).

In order to secure the homogeneity among regions, we selected the control area in South Chungcheong Province. Buyeo is in the same administrative district with Hongseong, representing a similar size of area. There is no asbestos mine in Buyeo; only one asbestos mine exists within 10 km radius, outside of its boundaries. The region is also less affected by naturally occurring asbestos (NOA). For these reasons, we selected Buyeo as the control area (Figure 1).

### 2.3. Study Population

ARDs usually have a latent period, and a sufficient exposure time is required for respiratory diseases and cancers to develop due to environmental asbestos exposure. To clarify the impact and causality of inhabitation around asbestos mines, individuals with less than five years of total residence in the exposed and control areas were excluded. Those who lived in both the exposed and control areas were also excluded. We constructed a dynamic cohort including individuals in the exposed and control areas for each target ARD. If individuals, who were in the exposed and control areas at the beginning of the cohort study, migrated to other areas outside the exposed and control areas and then returned, we resumed observations after the previous residence period.

### 2.4. Follow-Up Period

Since the NHID has been established since 2002 and the incidence of ARDs was monitored for individuals living in the exposed and control areas for more than five years, the follow-up period was from 2007 to 2018. We analyzed ARDs including cancers that occurred during the follow-up period. We excluded cases in which the same diseases were already diagnosed before enrollment in the cohort. In addition, we analyzed the incidence of cancers from 2007 to 2017 to calculate the standardized incidence ratio (SIR) because the incidence data on cancers in the KCCR were only published until 2017.

### 2.5. Target Diseases

We selected respiratory diseases, benign respiratory neoplasms, and cancers known to be associated with asbestos and analyzed the incidence of the diseases. The study subjects were considered to have specific diseases if they had more than two outpatient visits or more than one inpatient admission with the diagnosis of specified disease codes. The diseases of interest and their respective ICD-10 codes were as follows: asbestos-related respiratory diseases including asbestosis (J61), pneumoconiosis except asbestosis (J60, J62–J65), pleural plaque (J92), pleural effusion (J90–J91), pleurisy (R09.1), and COPD (J40–J44). The study subjects were considered to have benign neoplastic lung masses if they had more than one visit or admission under the ICD-10 codes of D02.2, D14.3, and D38.1. In the case of cancers, laryngeal cancer (C32), lung cancer (C33–C34), malignant mesothelioma (C45), and ovarian cancer (C56), for which there is sufficient evidence of their association with asbestos in the International Agency for Research on Cancer (IARC) classification, as well as pharyngeal cancer (C10–C13), stomach cancer (C16), colon cancer (C18), and rectal cancer (C19–C20), for which there is limited evidence of their association with asbestos in the IARC classification, were selected as asbestos-related cancers. In addition, esophageal cancer (C15) and renal cancer (C64), which have been reported to be associated with asbestos, were selected as asbestos-related cancers; however, no evidence level was specified in the IARC classification. The study subjects were considered to have these cancers if they had more than one outpatient visitor inpatient admission under the respective ICD-10 codes and the KNHIS code for rare and incurable diseases (V193).

### 2.6. Statistical Analysis

We conducted descriptive statistical analyses of age, sex, household income, and health behaviors (smoking and drinking). Age was based on the year of enrolment in the cohort. Household income was divided into quartiles according to income-based insurance contributions. Smoking and drinking status was based on information on health behaviors from the national health screening database. There was no information on smoking and drinking for more than 30% of the subjects because they had never received a national health screening or had not answered the questionnaire on health behaviors properly. In cases where there was a lack of information on smoking and drinking, most of them may be attributed to no health screening rather than simply missing data. We assumed that those who had never received health screening might be at risk; thus, we classified the subjects with no information on smoking and drinking into a separate category without excluding them. For drinking status, “heavy drinking” was defined as drinking seven glasses or more per day for men and five glasses or more per day for women at least twice per week. We also calculated the average follow-up period and person-years in both the exposed and control areas. We performed t-test and chi-square test to determine if there were differences in age, sex, household income, smoking and drinking status, and follow-up period between the exposed and control areas. We obtained the incidence rate of ARDs by calculating the person-years for each disease in both the exposed and control areas. We performed survival analysis using Cox’s proportional hazards model; unadjusted HRs, HRs adjusted for age, sex, household income, smoking status, and drinking status, and corresponding 95% confidence intervals (CIs) were calculated. Incidence rates were calculated for asbestosis, pleural plaques, and mesothelioma; unadjusted HRs, HRs adjusted for age, sex, household income level, smoking status, and drinking status, and corresponding 95% CIs were also calculated by stratification for these diseases. In addition, we calculated the SIRs and corresponding 95% CIs of the asbestos-related cancers stratified by sex in the exposed and control areas using national cancer incidence rates for each age group. SIRs were calculated using the indirect standardization method. All statistical analyses were performed using SAS ver. 9.4 software (SAS Institute, Cary, NC, USA). 

## 3. Results

### 3.1. Baseline Characteristics of Subjects

The average follow-up years of the subjects included in the study were around 10 years, and the follow-up period in the control area was slightly longer than that in the exposed area. The follow-up person-years in Hongseong (exposed area) and Buyeo (control area) were 1,011,874.6 and 909,433.5 person-years, respectively. The subjects in the exposed area had an average age of 40.9 years and were younger than those in the control area (43.5 years). In the exposed area, the proportion of subjects under 40 was 47.5%, which was higher than that in the control area (43.1%). On the other hand, the proportion of elderly subjects over 65 in the exposed area was 18.6%, which was less than that in the control area (22.7%). The sex ratio of men to women was almost 1:1, and there was no significant difference between the exposed and control areas. The proportions of the highest income quartile (Q4) and second-highest income quartile (Q3) in the exposed area were 30.1% and 27.7%, respectively, which were slightly higher than those in the control area (Q4 29.0%, Q3 26.1%). Around 30% of the subjects had never received a national health screening or had not answered the questionnaire on smoking status; thus, it was not known whether they smoked, and the proportion of subjects with missing data was slightly higher in the exposed group. More than one-third of the subjects also had missing drinking status data for the same reason, and the proportion of subjects with missing data was slightly higher in the exposed group. The proportions of current smokers and heavy drinkers in the exposed area were 13.9% and 8.8%, respectively, slightly higher than those in the control area (13.7% and 8.3%, respectively) (Table 1). 

### 3.2. Incidence Rates and Survival Analyses for ARDs

The incidence rate per 100,000 person-years of asbestosis was 66.02 in the exposed area, much higher than the rate (1.10) in the control area. The risk of asbestosis in the exposed area was around 60.8 times higher than that in the control area, which was statistically significant (crude HR 60.82, 95% CI = 32.57–113.55). The risk of asbestosis was also high even when adjusting for age, sex, household income, and smoking and drinking status (adjusted HR 65.40, 95% CI = 35.02–122.12). For pleural plaques, the incidence rate per 100,000 person-years was 5.32 in the exposed area, higher than the rate (1.54) in the control area. The risk of pleural plaques in the exposed area was around 3.5 times higher than that in the control area, which was statistically significant (crude HR 3.47, 95% CI = 1.93–6.25). Similarly, the risk of pleural plaques was significantly higher when other covariates were adjusted (adjusted HR 3.55, 95% CI = 1.96–6.41). For malignant mesothelioma, the incidence rate per 100,000 person-years was 0.89 in the exposed area, higher than the rate (0.55) in the control area. The risk of mesothelioma in the exposed area was higher than that in the control area; however, it was not significant even when covariates were adjusted (crude HR 1.62, 95% CI = 0.54–4.83; adjusted HR 1.83, 95% CI = 0.61–5.47). For colon cancer, the risk in the exposed area was not significant without adjustment (crude HR 1.02, 95% CI = 0.92–1.14); however, when covariates were adjusted, it was significantly higher in the exposed area (adjusted HR 1.13, 95% CI = 1.01–1.26). For pneumoconiosis except asbestosis (crude HR 0.52, 95% CI = 0.39–0.69; adjusted HR 0.57, 95% CI = 0.43–0.75), COPD (crude HR 0.78, 95% CI = 0.76–0.79; adjusted HR 0.80, 95% CI = 0.79– 0.82), and benign lung mass (crude HR 0.54, 95% CI = 0.49–0.60; adjusted HR 0.57, 95% CI = 0.52–0.63), their risks were significantly lower in the exposed area. Pleural effusion (crude HR 0.83, 95% CI = 0.73–0.95), rectal cancer (crude HR 0.83, 95% CI = 0.73–0.94), and lung cancer (crude HR 0.90, 95% CI = 0.82–0.99) showed significantly higher risks in the control area than in the exposed area without adjustment; however, they were no longer significant after adjusting for covariates. There was no significant difference in the risk of other diseases between the exposed and control areas (Table 2).

### 3.3. Stratification Analysis for Malignant Mesothelioma, Asbestosis, and Pleural Plaques

When stratified by sex, age, and household income, the risk of mesothelioma was significantly increased among men when other covariates were adjusted (adjusted HR 8.30, 95% CI = 1.04–66.63); however, other factors did not show statistical significance (Table 3).

For asbestosis, women (adjusted HR 89.75, 95% CI = 28.77–280.04) showed a stronger association. In age stratification analysis, asbestosis occurred only in the exposed area under the age of 40, showing an infinite estimate. However, the number of cases was small in the younger age groups; thus, older subjects showed the stronger association (adjusted HR 76.20, 95% CI = 31.51–184.23). In household income stratification analysis, in the lowest income group, there were 88 asbestosis cases in the exposed area, whereas there was no case in the control group; thus, the lowest income group showed the strongest association (Table 4).

For pleural plaques, women (adjusted HR 4.80, 95% CI = 1.83–12.54), the age group of 40–64 years (adjusted HR 4.21, 95% CI = 1.60–11.08), and the second highest income group (adjusted HR 9.77, 95% CI = 2.29–41.76) showed a stronger association (Table 5).

### 3.4. Age-Standardized Incidence Ratios of Asbestos-Related Cancers

In comparison with the overall population of South Korea with age standardization, the SIR of rectal cancer was significantly increased for both men and women in the exposed and control areas, and the ratio was slightly lower in the exposed area than in the control area. The SIRs of esophageal cancer and laryngeal cancer were significantly increased only for men in both the exposed and control areas, and laryngeal cancer had a slightly higher ratio in the exposed area than in the control area. For colon cancer, the SIR was significantly increased for men in both the exposed and control areas, and the ratio was higher in the exposed area; in contrast, the SIR was significantly increased for women only in the exposed area. For pharyngeal cancer, the SIR was significantly increased for men only in the exposed area; in contrast, the SIR was significantly increased for women only in the control area. For stomach cancer, lung cancer, and mesothelioma, the SIRs were significantly increased only for men in the exposed area. For ovarian and renal cancer, there was no significant increase in the SIRs in both the exposed and control areas (Table 6).

## 4. Discussion

We used the NHID to analyze the incidence of ARDs among individuals who had been living around abandoned asbestos mines (exposed area) and in the control area without abandoned asbestos mines for more than five years. The risk of asbestosis was much higher in the exposed area than in the control area. The risk of pleural plaques was not as high as that of asbestosis; however, the risk was significantly higher in the vicinity of abandoned asbestos mines than in the control area. Malignant mesothelioma showed a nearly two-fold increased risk in the vicinity of asbestos mines compared with the control area; however, this result was not statistically significant. When a separate analysis according to sex was conducted, the risk of mesothelioma among male residents was statistically significant. Among asbestos-related cancers, colon cancer showed a significantly increased risk of occurrence in the vicinity of asbestos mines compared with the control area when the confounders were adjusted. However, the effect size was slightly larger than 1.1; thus, the actual risk was not that high. Other asbestos-related cancers did not show a significant increase in occurrence in the vicinity of asbestos mines compared with the control area. However, when compared with the incidence of cancer in the overall population of South Korea, the incidences of colon and rectal cancer were significantly increased for both men and women near abandoned asbestos mines.

The significant increase in asbestosis and pleural plaques was consistent with previous findings showing the prevalence of asbestosis and pleural plaques in an epidemiologic survey conducted by the ME for residents living near asbestos mines. In the study by the ME at that time, the number of subjects was small, and there was no control group. On the other hand, our study was conducted on all residents living in Hongseong for more than five years, where many asbestos mines are located, and control areas without asbestos mines in the same province were selected based on the same criteria used for the exposed area.

Among ARDs, asbestoses follow a dose-response relationship and mainly occur at high concentrations of asbestos exposure [9]. However, pleural plaques may also occur at low concentrations of asbestos exposure [10]. In most surveys, pleural plaques are the most common radiological findings in individuals who were exposed to asbestos. A pleural plaque itself is harmless and is an objective sign of previous asbestos inhalation [11,12]. Pleural plaques are not precancerous lesions, and there is no evidence of increased risk of lung cancer or malignant mesothelioma due to the presence of pleural plaques; however, as a biomarker for asbestos exposure, they can help determine the magnitude of health damage caused by asbestos exposure [13].

In contrast to pleural plaques, the occurrence of asbestosis is not common in cases of environmental exposure to asbestos considering that it is caused by high levels of asbestos exposure [13,14]. However, an exposure assessment study by Camargo et al. found that there was a significant amount of asbestos exposure in the vicinity of asbestos mines in Canada [3]. In addition, in the study by Camus et al., the standardized mortality ratio (SMR) of asbestosis was significantly increased to 23.49 (95% CI = 2.64–84.83) among women living near two asbestos mines in Quebec [15]. These other studies suggested that it is possible to develop asbestosis due to environmental asbestos exposure near asbestos mines.

Hongseong, which was selected as an asbestos-exposed area, had nine asbestos mines, of which the Gwangcheon mine is the largest chrysotile mine in Asia until it was abandoned in 1984 [16]. The work environment measurement conducted by Moon is the only study at the time when the Gwangcheon mine was operational [17]. The asbestos fiber concentrations in the asbestos mine, the grinding plant, and a site as far as 2 m from the mining office were 0.092–0.385 f/cc, 2.671–5.966 f/cc, and 3.882 f/cc, respectively. The asbestos fiber concentration was higher in the sub-factory or around the mine than in the mine [17]. Since the mines were closed, most of the mines were left untouched, and asbestos was likely to be released around the abandoned mines for some time. Song et al. sampled and analyzed the soil in the vicinity of the Gyewol and Gwangcheon mines located in Hongseong from 2007 to 2008 and found a large amount of asbestos fibers [18]. Most of them are chrysotile fibers with some tremolite and actinolite fibers. However, the concentration of asbestos in the air around abandoned mines measured in 2010 was as low as 0.0007–0.0023 f/cc [19]. In the past, exposure to asbestos may be common; however, exposure to asbestos directly from abandoned asbestos mines is extremely rare these days. The high asbestos concentration in the soil in Hongseong may be attributed to NOA. In addition, activity-based sampling conducted in 2011 revealed that the concentration of asbestos fibers could exceed the exposure limit in certain activities such as agricultural activities and riding motorcycles [20]. Therefore, residents living near asbestos mines may be exposed to asbestos through other means. Both past exposure to asbestos mining and current exposure to NOA in the vicinity of abandoned asbestos mines should be considered in the analysis of the health effects of asbestos exposure.

In this study, when women lived in the asbestos-exposed areas, there was a higher risk of asbestosis and pleural plaques than in control areas. In general, occupational exposure to asbestos is more common among men than among women; however, for environmental exposure, women are exposed to asbestos to the same extent as men are [21]. Among the study subjects, men are more likely to be exposed to occupational asbestos, such as from working in asbestos mines in the past, whereas women are relatively less likely to be exposed; thus, the higher risks of asbestosis and pleural plaques for women might be attributed to environmental asbestos. However, the risk of mesothelioma was higher for men, and it is known that women around asbestos mines were involved in extracting asbestos during home craft production in the past [22]. Although occupational asbestos exposure in women cannot be excluded, the follow-up period in this cohort was from 2007, and as of 2007, the average age was in the early 40 s. Considering that as the import of raw asbestos began from 1960s, the mining had dwindled down with the last asbestos mine in Hongseong abandoned in 1984, it is unlikely that ever that many women could have occupational exposure at that time. Asbestosis is not easily distinguished from idiopathic pulmonary fibrosis. Therefore, the history of asbestos exposure and the presence of pleural plaques or pleural thickening are important in the diagnosis of asbestosis [23]. We defined disease occurrence based on the ICD-10 codes of the NHID; however, as it is difficult to diagnose asbestosis in radiological medicine, it is difficult to guarantee the reliability of the diagnosis. If residents living in Hongseong showed pulmonary fibrosis on radiographic imaging, asbestos exposure may be considered because they live near asbestos mines, and there could be a tendency to give a diagnosis of asbestosis. As a result, those diagnosed as having asbestosis in the exposed area might have been overdiagnosed.

Malignant mesothelioma can occur even with a relatively low exposure to asbestos. Personal interviews with 78 patients selected from 235 cases of malignant mesothelioma collected from 2001 to 2007 in the malignant mesothelioma surveillance system in South Korea revealed that 13 patients were exposed to environmental asbestos when living near asbestos mines or repairing slate buildings. Among women living near chrysotile mines in Quebec, Canada, the SMR of pleural mesothelioma was increased to 7.63 (95% CI = 3.06–15.73) [15], and in a cohort study of individuals who lived near crocidolite mines in Wittenoom, Australia, during their childhood, the SMR of mesothelioma was significantly increased to 88.71 (95% CI = 62.13–122.81) [24]. In our analysis, the incidence of malignant mesothelioma among residents near abandoned asbestos mines was nearly twice of that among residents in the control area; however, there was no statistical significance. For men, the risk of mesothelioma in the exposed area was nearly eight times higher than that in the control area, and it was statistically significant when adjusted for covariates. In addition, compared with the incidence in the overall population of men in South Korea, the SIR was significantly increased to 3.48, indicating the possibility of an increased risk of mesothelioma due to environmental exposure. However, for women, the risk of mesothelioma in the exposed area was lower than that in both the control area and the general population. It is highly possible that the risk was high only for men because of occupational exposure in the past; thus, cautious interpretation is needed. There are limited studies on the occurrence of lung cancer or death from lung cancer in the vicinity of asbestos mines, and the results are inconsistent. Although the mortality rate of lung cancer was not increased in a Canadian cohort study of women living near chrysotile mines in Quebec (SMR 0.99, 95% CI = 0.78–1.25) [15], in an Australian cohort study of women living near crocidolite mines in Wittenoom, the incidence of lung cancer was significantly increased (SIR 2.09, 95% CI = 1.34–2.84) [25]. However, there was no significant increase in lung cancer mortality in another cohort study of individuals who lived near crocidolite mines in Wittenoom during their childhood (SMR 1.89, 95% CI = 0.69–4.11) [24]. In our study, the incidence of lung cancer was not significantly different between those living near asbestos mines and those living in the control area. In comparison with the incidence in the overall population of men in South Korea, the SIR was significantly increased. However, the effect size was small (around 1.1); thus, the result may have limited practical implications. In general, the occurrence of lung cancer due to asbestos is known to be associated with exposure to a large amount of asbestos [26]. Therefore, the health effects due to environmental exposure in previous studies were inconsistent, and no significant increase was observed in this study.

When the incidences of asbestos-related cancers were compared with those in the general population of South Korea, the SIRs of colon and rectal cancer were significantly increased for both men and women. In the case of colorectal cancer, a significant increase in the incidence was also observed when compared with that in the control group. Although IARC classified the evidence for the association between asbestos and colorectal cancer as limited [27], the mortality risk of colorectal cancer was found to be significantly increased with occupational exposure in the most recent meta-analysis [28]. However, the occurrence of gastrointestinal (GI) cancer following environmental asbestos exposure has not been reported or investigated. Regarding the mechanism of colorectal cancer metastasis caused by asbestos, there is a hypothesis indicating that asbestos fibers penetrate into the intestines and increase the risk due to exposure to water contaminated with asbestos [29,30]. In cohort studies involving lighthouse keepers, the incidence of colorectal cancer was found to be significantly increased for the group exposed to drinking water contaminated with asbestos [31]. When water suppliers in the vicinity of Quebec’s Thetford chrysotile mines were surveyed in 1971, asbestos was detected in more than 80 of the 426 water suppliers, and the concentration of asbestos was as high as 140 million fibers per liter (MFL) [32]. In South Korea, the ME has been measuring asbestos contamination in the groundwater in the vicinity of asbestos mines annually since 2011, and the measurements were less than 7 MFL, the exposure standard for asbestos in drinking water [33].

In this study, the risk of pneumoconiosis except asbestosis, COPD, and benign lung mass were rather significantly lower in the exposed area. South Chungcheong Province had many coal mines as well as asbestos mines. In the past, 76 coal mines were in South Chungcheong Province, of which two were in Hongseong, asbestos-exposed area in present study, and 11 were in Buyeo, control area in present study [34]. These past coal mines are thought to have affected the risk of some respiratory diseases, including pneumoconiosis, COPD and lung mass.

This study has several limitations. First, the validation of the diagnosis for non-malignant respiratory diseases in the NHID was difficult. As we used the code for rare and incurable diseases, the validity of diagnosis was highly consistent with the statistical data of the KCCR but not non-malignant diseases. There may be discrepancies between the diagnoses in the NHID and diseases that patients had in reality due to the inherent nature of the claims data [35]. Second, the NHID lacked some information. It did not contain laboratory records and radiographic information, and smoking and drinking information in the database was incomplete because nearly 30% of the subjects had never received a national health check-up. Third, since the database was constructed in 2002, residential information before 2002 was not available. Considering the latent period of ARDs after asbestos exposure, the lack of past residential information might cause a misclassification for the exposed group. However, given that more than 70% of the study subjects continued to live in the area from 2002 to 2006 (Supplementary Table S1), it is likely that a large number of them also lived there before 2002. In addition, we could not differentiate the effect of occupational asbestos exposure because we did not know the occupational history of the subjects in the study. In the NHID, the code for the job and industry is provided for employee subscribers; however, it is impossible to identify asbestos-exposed jobs with this classification system. Furthermore, for regional insurance subscribers, the code of the job and industry is not provided. Lastly, we could not use the residential information of the subdivision (Eup-Myeon-Dong) of Hongseong due to the protection of personal information. Some of the residents in Hongseong, who were included in the exposed group, might live outside the 5 km radius of the asbestos mines; thus, the effect of asbestos exposure for the exposed area might be reduced, and the causal relationship could have been weakened. Moreover, because of this limitation, we could not analyze the effect of the distance from asbestos mines.

Despite these limitations, this study is a well-designed epidemiological study for ARDs compared with previous studies in South Korea. The sample size was large, an appropriate control group was selected, and objective risk estimates were calculated for asbestos-related cancers through comparison with KCCR data. In addition, as we used the code for rare and incurable diseases, the accuracy and reliability of the diagnosis were high and almost consistent with the actual cancer incidence. The analysis of big data using the NHID is one of the approaches used in epidemiological studies to accurately determine the degree of environmental asbestos exposure. Although there are limitations such as the restrictions of claims data and the lack or absence of information, significant causality may be inferred by showing consistent results for asbestosis and pleural plaques.

## 5. Conclusions

In our study using the NHID, the risks of asbestosis and pleural plaques were consistently higher among residents living near abandoned asbestos mines compared with those in the control area. The risk of malignant mesothelioma in the exposed area was not significant; however, when limited to male residents, the risk was significantly increased. For GI cancer, there was a significant increase in the risk of colon cancer in the exposed area compared with the control area, and when compared with the general population of South Korea, a significant increase in the risks of both colon and rectal cancer was observed. Although the effects of occupational asbestos exposure cannot be excluded and the interpretation of the results should be cautious due to the limitations of the NHID, our study demonstrated that environmental asbestos exposure could cause ARDs.

## Figures and Tables

**Table 1:** Baseline characteristics of the subjects.

Characteristics	Exposed Area (Hongseong) N = 104,198	Control Area (Buyeo) N = 90,640	*p*-Value
Mean age (years)	40.9 ± 22.5	43.5 ± 22.9	<0.0001
Age (years)			<0.0001
<20	23,335 (22.4)	17,844 (19.7)	
20–39	26,100 (25.1)	21,216 (23.4)	
40–64	35,361 (33.9)	31,034 (34.2)	
≥65	19,402 (18.6)	20,546 (22.7)	
Sex			0.1857
Men	51,764 (49.7)	45,301 (50.0)	
Women	52,434 (50.3)	45,339 (50.0)	
Household income			<0.0001
Q1 (lowest)	22,107 (21.5)	22,137 (24.8)	
Q2	21,180 (20.6)	17,943 (20.1)	
Q3	28,566 (27.7)	23,328 (26.1)	
Q4 (highest)	30,938 (30.1)	25,965 (29.0)	
Smoking			<0.0001
Never smoker	48,314 (46.4)	41,712 (46.0)	
Ex-smoker	7835 (7.5)	8763 (9.7)	
Current smoker	14,530 (13.9)	12,405 (13.7)	
Missing *	33,500 (32.2)	27,758 (30.6)	
Alcohol			<0.0001
Non-drinking	41,075 (39.4)	36,763 (40.6)	
Normal drinking	16,395 (15.7)	14,406 (15.9)	
Heavy drinking **	9137 (8.8)	7524 (8.3)	
Missing *	37,572 (36.1)	31,945 (35.2)	
Average follow-up years	9.7 ± 3.5	10.03 ± 3.3	<0.0001
Person-year	1,011,874.6	909,433.5	

* Those had never received a national health screening or had not answered the questionnaire of health behaviors ** Heavy drinking: consumption more than 7 glasses per day (men) or 5 glasses per day (women) at least twice per week.

**Table 2:** Incidence rates and hazard ratios of asbestos-related diseases in the exposed and control areas.

Diseases	No. of Cases	Person-Years	Incidence *	HR ** (95% CI)	
				Crude	Adjusted
Asbestosis (J61)					
Control area	10	909,433.5	1.10	1	1
Exposed area	668	1,011,874.6	66.02	60.82 (32.57–113.55)	65.40 (35.02–122.12)
Pneumoconiosis (except asbestosis; J60, J62–J65)					
Control area	133	907,699.4	14.65	1	1
Exposed area	77	1,014,133.8	7.59	0.52 (0.39–0.69)	0.57 (0.43–0.75)
Pleural effusion (J90–J91)					
Control area	490	906,782.6	54.04	1	1
Exposed area	454	1,012,520.8	44.84	0.83 (0.73–0.95)	0.94 (0.82–1.06)
Pleural plaques (J92)					
Control area	14	909,311.8	1.54	1	1
Exposed area	54	1,014,573.7	5.32	3.47 (1.93–6.25)	3.55 (1.96–6.41)
COPD (J40-J44)					
Control area	18184	631,752.2	2878.34	1	1
Exposed area	17355	777,513.3	2232.12	0.78 (0.76–0.79)	0.80 (0.79–0.82)
Pleurisy (R09.1)					
Control area	18	909,302.8	1.98	1	1
Exposed area	15	1,014,747.1	1.48	0.74 (0.37–1.47)	0.81 (0.41–1.60)
Benign lung mass (D02.2, D14.3, D38.1)					
Control area	1018	900,904.0	113.00	1	1
Exposed area	617	1,009,735.8	61.11	0.54 (0.49–0.60)	0.57 (0.52–0.63)
Pharyngeal cancer (C10–C13)					
Control area	37	909,241.8	4.07	1	1
Exposed area	48	1,014,660.4	4.73	1.17 (0.76–1.79)	1.29 (0.84–1.99)
Esophageal cancer (C15)					
Control area	101	909,043.7	11.11	1	1
Exposed area	105	1,014,472.6	10.35	0.93 (0.71–1.22)	1.04 (0.79–1.38)
Stomach cancer (C16)					
Control area	1018	901,648.6	112.90	1	1
Exposed area	1100	1,006,940.7	109.24	0.97 (0.89–1.05)	1.04 (0.98–1.16)
Colon cancer (C18)					
Control area	598	905,936.9	66.01	1	1
Exposed area	681	1,010,434.7	67.40	1.02 (0.92–1.14)	1.13 (1.01–1.26)
Rectal cancer (C19–C20)					
Control area	496	905,724.1	54.76	1	1
Exposed area	458	1,011,198.6	45.29	0.83 (0.73–0.94)	0.90 (0.79–1.02)
Laryngeal cancer (C32)					
Control area	53	909,148.1	5.83	1	1
Exposed area	63	1,014,448.5	6.21	1.07 (0.74–1.54)	1.14 (0.79–1.64)
Lung cancer (C33–C34)					
Control area	866	907,004.2	95.48	1	1
Exposed area	871	1,012,289.2	86.04	0.90 (0.82–0.99)	1.02 (0.93–1.12)
Mesothelioma (C45)					
Control area	5	909,463.6	0.55	1	1
Exposed area	9	1,014,844.6	0.89	1.62 (0.54–4.83)	1.83 (0.61–5.47)
Ovarian cancer (C56)					
Control area	77	908,952.0	8.47	1	1
Exposed area	64	1,014,298.7	6.31	0.75 (0.54–1.04)	0.79 (0.56–1.10)
Renal cancer (C56)					
Control area	102	908,774.9	11.22	1	1
Exposed area	104	1,014,271.5	10.25	0.92 (0.70–1.21)	1.03 (0.78–1.35)

* Incidence rate per 1,000 person-years ** HR: hazard ratio.

**Table 3:** Incidence rates and hazard ratios of mesothelioma stratified by sex, age, and household income.

Variables	Exposed Area			Control Area			HR ** (95% CI)	
	No. of Cases	Person-Years	Incidence *	No. of Cases	Person-Years	Incidence *	Unadjusted	Adjusted
Sex								
Men	8	501,194.0	1.60	1	452,184.3	0.22	7.20 (0.90–57.56)	8.30 (1.04–66.63)
Women	1	513,650.6	0.19	4	457,279.2	0.87	0.23 (0.03–2.01)	0.25 (0.03–2.23)
Age (years)								
<20	0	218,039.2	0.00	0	176,845.9	0.00	‒	‒
20–39	0	266,223.8	0.00	0	224,046.5	0.00	‒	‒
40–64	4	355,563.6	1.12	1	323,485.6	0.31	3.69 (0.41–33.04)	4.03 (0.45–36.21)
≥65	5	174,917.9	2.86	4	185,085.6	2.16	1.32 (0.36–4.93)	1.21 (0.33–4.53)
Household income								
Q1 (lowest)	3	208,783.9	1.44	1	216,561.5	0.46	3.06 (0.32–29.40)	3.31 (0.34–31.84)
Q2	0	206,732.4	0.00	0	177,763.9	0.00	‒	‒
Q3	2	281,235.1	0.71	1	237,052.2	0.42	1.66 (0.15–18.29)	1.82 (0.16–20.12)
Q4 (highest)	4	305,751.3	1.31	3	266,423.6	1.13	1.18 (0.27–5.28)	1.34 (0.30–6.03

* Incidence rate per 1,000 person-years ** HR: hazard ratio.

**Table 4:** Incidence rates and hazard ratios of asbestosis stratified by sex, age, and household income.

Variables	Exposed Area			Control Area			HR * (95% CI)	
	No. of Cases	Person-Years	Incidence **	No. of Cases	Person-Years	Incidence **	Unadjusted	Adjusted 1
Sex								
Men	391	499,450.4	78.29	7	452,155.3	1.55	51.03 (24.19–107.66)	53.30 (25.24–112.55)
Women	277	512,424.2	54.06	3	457,278.2	0.66	83.57 (26.79–260.69)	89.75 (28.77–280.04)
Age (years)								
<20	1	218,032.7	0.46	0	176,845.9	0.00	Infinite	Infinite
20–39	4	266,211.7	1.50	0	224,046.5	0.00	Infinite	Infinite
40–64	294	354,525.7	82.93	5	323,475.8	1.55	54.73 (22.61–132.46)	51.95 (21.46–125.79)
≥65	369	173,104.5	213.17	5	185,065.4	2.70	79.09 (32.73–191.14)	76.20 (31.51–184.23)
Household income								
Q1 (lowest)	88	208,406.6	42.23	0	216,562.6	0.00	Infinite	Infinite
Q2	126	206,140.4	61.12	4	177,742.5	2.25	27.37 (10.12–74.06)	29.42 (10.87–79.66)
Q3	224	280,268.1	79.92	3	237,049.4	1.27	64.20 (20.55–200.55)	71.54 (22.89–223.60)
Q4 (highest)	218	304,760.5	71.53	3	266,416.6	1.13	64.34 (20.59–201.05)	73.75 (23.59–230.53)

* HR: hazard ratio ** Incidence rate per 1000 person-years.

**Table 5:** Incidence rates and hazard ratios of pleural plaques stratified by sex, age, and household income.

Variables	Exposed Area			Control Area			HR * (95% CI)	
	No. of Cases	Person-Years	Incidence **	No. of Cases	Person-Years	Incidence **	Unadjusted	Adjusted
Sex								
Men	28	501,068.6	5.59	9	452,065.8	1.99	2.83 (1.34–6.00)	2.94 (1.37–6.32)
Women	26	513,505.1	5.06	5	457,245.9	1.09	4.65 (1.79–12.10)	4.80 (1.83–12.54)
Age (years)								
<20	0	218,039.2	0.00	0	176,845.9	0.00	–	–
20–39	0	266,203.8	0.00	1	224,045.0	0.45	–	–
40–64	26	35,548.6	73.14	5	323,537.9	1.55	4.78 (1.83–12.44)	4.21 (1.60–11.08)
≥65	28	174,782.0	16.02	8	184,983.0	4.32	3.70 (1.69–8.13)	3.57 (1.62–7.85)
Household income								
Q1 (lowest)	2	208,775.6	0.96	1	216,525.9	0.46	2.07 (0.19–22.78)	2.14 (0.19–23.61)
Q2	7	206,694.0	3.39	4	177,721.2	2.25	1.51 (0.44–5.16)	1.69 (0.49–5.82)
Q3	21	281,120.7	7.47	2	237,034.9	0.84	8.88 (2.08–37.86)	9.77 (2.29–41.76)
Q4 (highest)	21	305,652.7	6.87	7	266,367.3	2.63	2.64 (1.12–6.21)	3.00 (1.27–7.09)

* HR: hazard ratio ** Incidence rate per 1000 person-years.

**Table 6:** Standardized incidence ratios of asbestos-related cancer in 2007–2017.

Cancer	Exposed Area			Control Area		
	Obs *	Exp *	SIR *	Obs *	Exp *	SIR *
Pharyngeal cancer (C10–C13)						
Men	39	21.8	1.79 (1.27–2.45)	26	21.8	1.20 (0.78–1.75)
Women	5	3.5	1.44 (0.47–3.37)	9	3.3	2.73 (1.25–5.17)
Esophageal cancer (C15)						
Men	93	66.4	1.40 (1.13–1.72)	90	68.3	1.32 (1.06–1.62)
Women	7	6.6	1.07 (0.43–2.20)	8	6.8	1.17 (0.51–2.31)
Stomach cancer (C16)						
Men	716	580.0	1.23 (1.15–1.33)	622	582.2	1.07 (0.99–1.16)
Women	299	288.3	1.04 (0.92–1.16)	312	290.5	1.07 (0.96–1.20)
Colon cancer (C18)						
Men	389	258.8	1.50 (1.36–1.66)	329	262.9	1.25 (1.12–1.39)
Women	246	193.2	1.27 (1.12–1.44)	220	198.2	1.11 (0.97–1.27)
Rectal cancer (C19–C20)						
Men	278	208.5	1.33 (1.18–1.50)	304	208.5	1.46 (1.30–1.63)
Women	150	126.8	1.18 (1.00–1.39)	156	128.2	1.22 (1.03–1.42)
Laryngeal cancer (C32)						
Men	60	33.3	1.80 (1.37–2.32)	49	34.1	1.44 (1.06–1.90)
Women	6	2.25	2.66 (0.98–5.80)	4	2.3	1.71 (0.47–4.37)
Lung cancer (C33–C34) **						
Men	562	511.1	1.10 (1.01–1.19)	553	531.7	1.04 (0.96–1.13)
Women	234	212.1	1.10 (0.97–1.25)	222	220.4	1.01 (0.88–1.15)
Mesothelioma (C45) **						
Men	8	2.3	3.48 (1.50–6.85)	1	2.3	0.43 (0.01–2.39)
Women	1	1.17	0.85 (0.02–4.76)	3	1.2	2.55 (0.53–7.45)
Ovarian cancer (C56) **						
Women	58	55.1	1.05 (0.80–1.36)	62	51.8	1.20 (0.92–1.54)
Renal cancer (C56) **						
Men	60	75.4	0.80 (0.61–1.02)	55	73.4	0.75 (0.56–0.97)
Women	34	35.4	0.96 (0.67–1.34)	34	34.9	0.97 (0.67–1.36)

* Obs: observed number, Exp: expected number, SIR: Standardized incidence ratio ** Classified as sufficient evidence of their association with asbestos by IARC.

## Data Availability

The data used in this study is available from the Korean National Health Insurance Service (KNHIS). However, this data requires deliberation and approval by the KNHIS and can be assessed only through the local intra-network of KNHIS.

## Supplementary Materials

The following are available online at https://www.mdpi.com/1660-4601/18/3/875/s1, Table S1: Distribution of years enrolled in exposed and control areas.
