# Supplementary material for: Incidence of Cancer and Asbestos-Related Diseases among Residents Living near Abandoned Asbestos Mines in South Korea: A Retrospective Cohort Study Using National Health Insurance Database
Source: Int J Environ Res Public Health. 2021 Jan 20;18(3):875. doi: 10.3390/ijerph18030875 (PMC7908467; doi:10.3390/ijerph18030875)
Supplement: Supplementary file 1 [file ijerph-18-00875-s001.pdf]

**Supplementary Table S1.** Distribution of years enrolled in exposed and control areas.

| Year | Exposed area (Hongseong) | Control area (Buyeo) |
|---|---|---|
| 2007 | 70,952 (68.1) | 67,170 (74.1) |
| 2008 | 4,749 (4.6) | 4,280 (4.7) |
| 2009 | 4,041 (3.9) | 2,938 (3.2) |
| 2010 | 3,340 (3.2) | 2,192 (2.4) |
| 2011 | 2,863 (2.7) | 2,116 (2.3) |
| 2012 | 2,823 (2.7) | 1,913 (2.1) |
| 2013 | 2,666 (2.6) | 1,898 (2.1) |
| 2014 | 2,540 (2.4) | 1,615 (1.8) |
| 2015 | 2,420 (2.3) | 1,697 (1.9) |
| 2016 | 2,427 (2.3) | 1,578 (1.7) |
| 2017 | 2,565 (2.5) | 1,605 (1.8) |
| 2018 | 2,812 (2.7) | 1,638 (1.8) |
| Total | 104,198 (100.0) | 90,640 (100.0) |
